# In vivo cooling‐induced intracellular Ca^2+^ elevation and tension in rat skeletal muscle

**DOI:** 10.14814/phy2.14921

**Published:** 2021-07-10

**Authors:** Ryo Takagi, Ayaka Tabuchi, David C. Poole, Yutaka Kano

**Affiliations:** ^1^ Graduate School of Informatics and Engineering University of Electro‐Communications Tokyo Japan; ^2^ Research Fellowship for Young Scientists Japan Society for the Promotion of Science Tokyo Japan; ^3^ Department of Anatomy & Physiology and Kinesiology Kansas State University Manhattan Kansas USA; ^4^ Center for Neuroscience and Biomedical Engineering University of Electro‐Communications Tokyo Japan

**Keywords:** caffeine, calcium‐induced calcium release, rapid cooling contracture, temperature

## Abstract

It is an open question as to whether cooling‐induced muscle contraction occurs in the in vivo environment. In this investigation, we tested the hypotheses that a rise in intracellular Ca²⁺ concentration ([Ca²⁺]i) and concomitant muscle contraction could be evoked in vivo by reducing muscle temperature and that this phenomenon would be facilitated or inhibited by specific pharmacological interventions designed to impact Ca²⁺‐induced Ca²⁺‐release (CICR). Progressive temperature reductions were imposed on the spinotrapezius muscle of Wistar rats under isoflurane anesthesia by means of cold fluid immersion. The magnitude, location, and temporal profile of [Ca²⁺]i were estimated using fura‐2 loading. Caffeine (1.25–5.0 mM) and procaine (1.6–25.6 mM) loading were applied in separatum to evaluate response plasticity by promoting or inhibiting CICR, respectively. Lowering the temperature of the muscle surface to ~5°C produced active tension and discrete sites with elevated [Ca²⁺]i. This [Ca²⁺]i elevation differed in magnitude from fiber to fiber and also from site to site within a fiber. Caffeine at 1.25 and 5.0 mM reduced the magnitude of cooling necessary to elevate [Ca²⁺]i (i.e., from ~5°C to ~8 and ~16°C, respectively, both *p* < 0.05) and tension. Conversely, 25.6 mM procaine lowered the temperature at which [Ca²⁺]i elevation and tension were detected to ~2°C (*p* < 0.05). Herein we demonstrate the spatial and temporal relationship between cooling‐induced [Ca²⁺]i elevation and muscle contractile force in vivo and the plasticity of these responses with CICR promotion and inhibition.

## Graphical abstract

We observed a muscle temperature threshold (approximately 5°C) for in vivo elevation of calcium ion and active tension development in response to cooling. This temperature threshold exhibited highly plastic behavior in response to pharmacological promotion or inhibition of sarcoplasmic reticulum calcium‐induced calcium release.

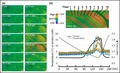

## 1 INTRODUCTION

Temperature is one of the most important environmental factors that directly affects biological reactions. In skeletal muscle, it has long been known that decreased temperature induces muscle contraction (Conway & Sakai, 1960; Sakai, 1986) and the contraction is observed in vitro at 4°C. In particular, Conway and Sakai found that lowering the temperature of turtle and frog skeletal muscle reduces the concentrations of caffeine or potassium sulfate required to produce contracture (Conway & Sakai, 1960). Furthermore, subsequently returning the temperature toward “normothermic” induces relaxation and this cyclical behavior was found to be reversible repeatedly with further cooling‐warming cycles. It is now recognized that this cooling‐induced contracture is caused by elevation of the intracellular Ca²⁺ concentration ([Ca²⁺]i) (Kurihara et al., 1984), and also that cooling increases tension in chemically skinned rabbit extensor digitorum longus muscle (EDL) fibers (Sudo et al., 1987) and in rat skeletal muscle where cold‐induced tension generation in the slow‐twitch soleus far exceeds that in the fast‐twitch EDL (Hill, 1972) in vitro.

Induction of the cooling response is thought to be caused by increased sarcoplasmic reticulum (SR) Ca²⁺ release in conjunction with decreased SR Ca²⁺ reuptake (Horiuti, 1988). In fact, SR Ca²⁺‐ATPase (SERCA) activity in SR isolated from frog skeletal muscle is reduced in a temperature‐dependent manner (Dode et al., 2001). Thus, the increased [Ca²⁺]i resulting from cooling‐induced enhancement of SR Ca²⁺ efflux subsequently potentiates the net SR Ca²⁺ release (i.e., Ca²⁺‐induced Ca²⁺release, CICR) and further elevates [Ca²⁺]i. This mechanism is consistent with the observation that, in single skeletal muscle fibers pre‐treated with low concentration caffeine, procaine, which suppresses CICR (Klein et al., 1992), almost completely abolishes the cooling‐induced [Ca²⁺]i increase (Kurihara et al., 1984) and associated tension development (Konishi et al., 1985).

Although the phenomenon during cooling has been valuable physiologically in vitro to demonstrate SR‐Ca²⁺ release, it has not, to our knowledge, been validated in the more complex in vivo environment in mammalian muscle where neurovascular control, muscle oxygenation, and myocyte energetics remain relatively unperturbed. Moreover, it is pertinent that the muscle system selected for this investigation is a close analog of the human quadriceps insofar as it possesses a mosaic of fast‐ and slow‐twitch fiber types (Delp & Duan, 1996) and similar oxidative capacity (Leek et al., 2001). Specifically, we tested the hypotheses that imposed cooling in mammalian muscle in vivo would: 1. increase [Ca²⁺]i causing contraction and elevating muscle tension production, and 2. that this process would be potentiated pharmacologically by a CICR promotor (caffeine) and constrained by CICR inhibition (procaine). Understanding the mechanisms of cooling‐induced Ca²⁺ dynamics in skeletal muscle in vivo will be of great value in developing new approaches to bring about muscle adaptation.

## 2 MATERIALS AND METHODS

### 2.1 Animals

Seven 10‐ to 11‐week‐old male Wistar rats (Japan SLC) were maintained under a 12:12‐h light–dark cycle and allowed ad libitum access to food and water throughout the experiments. This study was approved by the University of Electro‐Communications Institutional Animal Care and Use Committee (#2018‐30).

### 2.2 Muscle preparation for in vivo imaging

The muscle preparation for [Ca²⁺]i imaging was performed as previously reported (Sonobe et al., 2008). Specifically, the right spinotrapezius muscle was exteriorized gently without disruption of the principal neural and vascular pathways (Bailey et al., 2000; Kindig & Poole, 1998; Kindig et al., 2002; Poole et al., 1997). Careful access was obtained via a midline incision through the skin starting at the lower cervical level and extending caudally to the upper lumbar vertebral level under 0.8 L/min of 2.0 vol% isoflurane anesthesia with 0.1 L/min of 100 vol% oxygen. After exposure, the muscle was superfused with Krebs–Henseleit buffer [KHB (in mM); 132 NaCl, 4.7 KCl, 21.8 NaHCO₃, 2 MgSO₄, and 2CaCl₂] equilibrated with 95% N₂‐5% CO₂ and adjusted to pH 7.4, to ensure that the muscle was not subjected to high atmospheric oxygen pressures or desiccation. Subsequently, the spinotrapezius distal end (insertion) was detached and connected to a strain gauge‐linked motor device (RU‐72 model: Motomura Systems), by means of a custom‐made lightweight horseshoe manifold, and incubated in 40 µM fura‐2‐AM/KHB solution for 60 min before being rinsed with KHB to remove non‐loaded fura‐2‐AM. The muscle was then immersed in KHB adjusted to 30 ± 0.5°C and 340/380‐nm wavelength excitation light (200 ms, respectively) delivered using appropriate fluorescent filters. Pairs of fluorescence images were captured by a CMOS camera (ORCA‐Flash4.0; Hamamatsu Photonics, Hamamatsu) using image‐capture software (NIS‐Elements Advanced Research; Nikon) in no‐delay mode (approximately once every 1.5 s) through the 510‐nm emission wavelength filter for ratiometric estimation of [Ca²⁺]iwithout need for any additional processing. This study did not take steps to quantify the actual [Ca²⁺]i from that ratio value.

### 2.3 Rapid cooling‐induced contraction with and without caffeine or procaine

**FIGURE 1:**
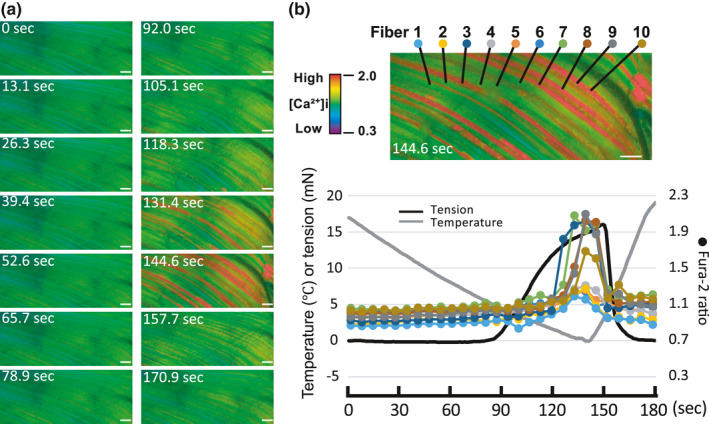
In vivo cooling‐induced [Ca²⁺]i elevation and contraction in rat spinotrapezius. Representative changes of [Ca²⁺]i and tension across a cooling‐rewarming cycle (i.e., ~20–0–20°C) over 180 s. The temporal profile of fura‐2 ratio responses are shown sequentially and labeled for time in the left side two panel photomicrograph columns (a). The fura‐2 ratio values (scale given) were averaged over a fixed area in 10 muscle fibers each of which is color‐coded and corresponds between the upper right photomicrograph and the lower graph (b). Bars = 100 µm

**FIGURE 2:**
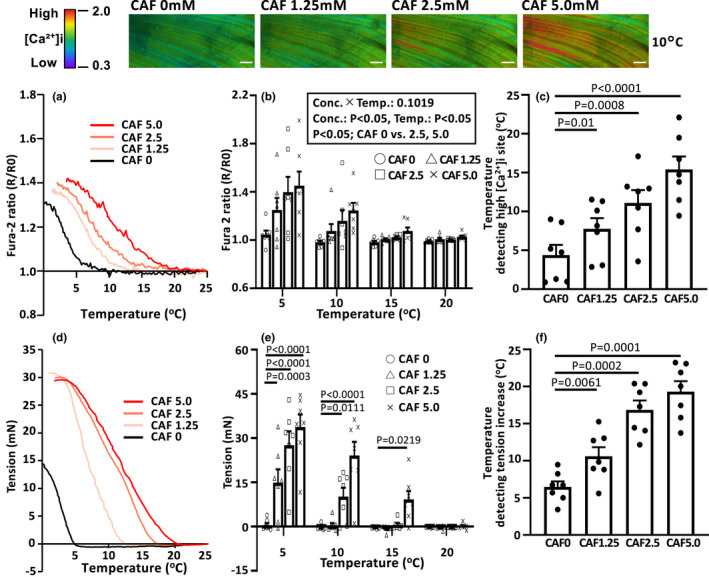
Potentiating effect of caffeine on cooling‐induced [Ca²⁺]i elevation and tension. Representative photomicrographs at 10°C in muscle fibers loaded with fura‐2 are depicted for increasing caffeine concentration from left to right (top panels). The left‐hand graph is a representative example of the overall average fura‐2 ratio (a) or absolute tension increase (d) plotted as a function of temperature change for each caffeine concentration. The center graphs show the fura‐2 ratio (b) and tension (e) for each caffeine concentration at 5, 10, 15, and 20°C. In the graphs at right, the temperature threshold at which high [Ca²⁺]i (c) and tension (f) increase are detected is demonstrated to increase progressively with higher caffeine concentration relative to control (i.e., 0 mM; *n* = 7 muscles). *p* < 0.05 was considered statistically significant. Values are means ± *SEs*. *CAF*, caffeine group. Bars = 100 µm

**FIGURE 3:**
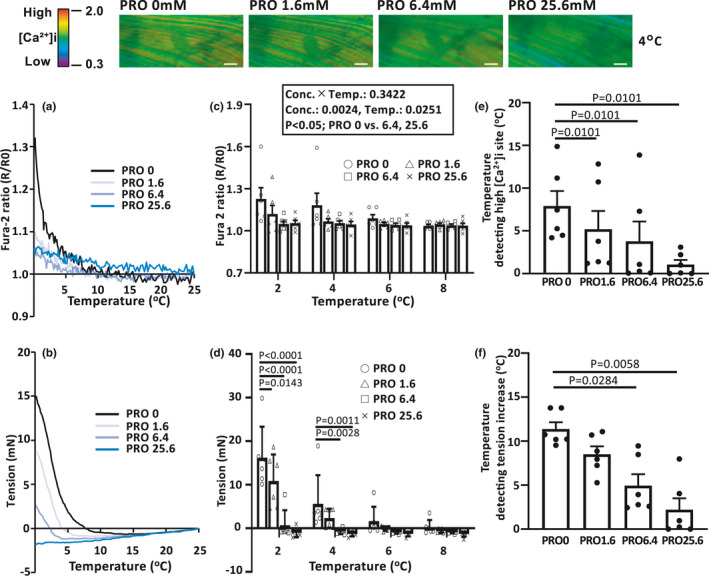
Inhibitory effect of procaine on cooling‐induced [Ca²⁺]i elevation and tension. Representative photomicrographs at 4°C in muscle fibers loaded with fura‐2 are depicted for increasing procaine concentration (left to right; top panels). The left‐hand graph is a representative example of the overall average fura‐2 ratio (a) or absolute tension increase (b) plotted as a function of temperature change for each procaine concentration. The center graphs show the fura‐2 ratio (c) and tension (d) for each procaine concentration at 2, 4, 6, and 8°C. In the graphs at right, the temperature threshold at which high[Ca²⁺]i (e) and tension (f) increase are detected is demonstrated to decrease progressively with higher procaine concentration relative to control (i.e., 0 mM; *n* = 6 muscles). *p* < 0.05 was considered statistically significant. Values are means ± *SEs*. *PRO*, procaine group. Bars = 100 µm

The temperature of the muscle immersion solution was monitored with a precision temperature probe (BAT‐10: Physitemp Instruments) and lowered at the maximum output of an electronic cooler (HMC‐12W‐0100: Hayashi‐Repic) located under the muscle, before being returned to the initial temperature as seen in Figure 1. In order to avoid the risk of adverse effects (irreversible muscle damage) from extremely low and non‐therapeutically relevant temperatures, the immersion fluid temperature was carefully controlled and monitored so that it did not fall below 0°C. After recording the contraction under the KHB solution immersion conditions (i.e., control, “CAF 0” in Figure 2), the KHB solution was replaced with caffeine in KHB at stepped concentrations between 1.25 and 5.0 mM. After 5 min equilibration time at each concentration, the temperature was controlled cyclically as above. Following the final caffeine concentration (i.e., 5.0 mM), the muscles were washed three times with KHB and at least 10 min was allowed to elapse before the procaine‐loading experiments were performed. As for the caffeine conditions, “PRO 0” in the procaine‐loading experiments in Figure 3 refers to the state after caffeine was washed out and serves as a timed control for the procaine experiments. The KHB solution was loaded with stepped increases of procaine concentration between 1.6 and 25.6 mM. The fura‐2 ratio and tension changes with varied temperature are referenced to the standard temperature of 25°C which represents a temperature at which no cooling response was evident herein under any conditions. For clarity of presentation, we compared the temperatures at which the tension begins to fully increase as the temperature decreases and operationally defined high [Ca²⁺]i sites as those with the ratio (340/380 nm) that are at least 2 *SD* higher than the average of all observed sites and that propagate with decreasing temperature.

### 2.4 Statistics

The differences associated with changes of temperature and caffeine or procaine concentration, were examined using a two‐way analysis of variance (ANOVA) followed by Dunnett's multiple comparisons test. The differences by concentration were examined using a one‐way ANOVA followed by Holm–Sidak's multiple comparisons test. Values of *p* < 0.05 were considered statistically significant.

## 3 RESULTS

### 3.1 In vivo [Ca²⁺]i imaging and tension during cooling‐induced contraction

The Supporting Information Video 1 (https://doi.org/10.6084/m9.figshare.13686619.v1) shows the [Ca²⁺]i dynamics during the cooling‐induced (i.e., 20°C–~0°C) contraction and subsequent relaxation (~0°C–20°C) cycle (at 4× actual speed) for the same muscle as presented in Figure 1. In addition, Figure 1b highlights the temperature‐induced fura‐2 ratio kinetics within the selected area across 10 muscle fibers simultaneously with tension changes during contraction and relaxation across that 20°C–~0°C–20°C cycle. Notice that the increase in the fura‐2 ratio is site‐specific and heterogeneous within and among myocytes and appears to be propagated.

### 3.2 Sensitization of muscle to cooling‐induced contraction by caffeine

The upper panels in Figure 2 present typical fura‐2 ratio images in response to 10°C cooling across the range of increasing caffeine concentrations (0–5.0 mM, left to right) imposed. No high [Ca²⁺]i sites were observed at 0 mM caffeine (Control), but more of these high [Ca²⁺]i sites were identified as the caffeine concentration was increased. This effect is demonstrated as a function of decreased temperature moving from right‐to‐left in Figure 2a,b. There was a striking elevation in the fura‐2 ratio at progressively higher temperatures as the caffeine concentration was increased until, in the extreme, at 5 mM caffeine concentration there were high [Ca²⁺]i sites evident at temperatures ~16°C (Figure 2c). This potentiation of the cooling effect by increasing caffeine concentration is also clear from the marked rightward shift of the elevated fura‐2 ratio curve plotted against temperature (Figure 2a). Figure 2d–f present the tension responses and notice the potentiation of the cooling‐induced tension increase as caffeine concentration is progressively raised (Figure 2d) such that at 5.0 mM caffeine concentration tension begins to increase sharply below ~20°C compared with <5°C for the control (0 mM caffeine concentration) condition. The systematically stepped increase in tension with progressively elevated caffeine concentration at 5°C (Figure 2e) is an especially striking exemplar of the caffeine‐induced sensitization of the cooling effect. Finally in the rightmost graphs, there is a compelling correspondence between how increasing caffeine concentration from left‐to‐right systematically raises the temperature threshold for detection of both high [Ca²⁺]i (Figure 2c) and tension (Figure 2f). Specifically, compared with the control (0 mM caffeine concentration) thresholds of 4.4 ± 1.2°C for [Ca²⁺]i and 6.5 ± 0.7°C for tension the thresholds for the caffeine concentration of 1.25mM were 7.8 ± 1.3°C for [Ca²⁺]i and 10.6 ± 1.1°C for tension, respectively. At 2.5 mM caffeine concentration the corresponding values were 11.1 ± 1.5°C for [Ca²⁺]i and 16.8 ± 1.2°C for tension, and at 5.0 mM caffeine concentration, 15.4 ± 1.5°C for [Ca²⁺]i and 19.3 ± 1.3°C for tension.

### 3.3 Inhibitory effect of procaine on cooling‐induced contraction

The upper panels in Figure 3 denote representative fura‐2 ratio images in response to 4°C cooling across the range of increasing procaine concentrations from 0 to 25.6 mM (left‐to‐right). Note that high [Ca²⁺]i sites were no longer detected at a procaine concentration of 1.6 mM for this particular muscle. In Figure 3a,b, note that the overall response to increasing procaine concentration is diametrically opposite to that for caffeine observed previously with a shift to the left in response to systematically elevating procaine concentration. Figure 3c,d further emphasize the inhibitory effect of rising procaine concentration on the cooling‐induced fura‐2 ratio and tension responses. For clarity, we have focused on the temperature range from 2 to 8°C to best reveal the inhibitory effect of procaine on the cooling response. For instance, at 2°C compared with the 0 mM procaine concentration control, the fura‐2 ratio was significantly lower at procaine concentrations of 6.4 and 25.6 mM whereas the tension was significantly reduced at procaine concentrations of 1.6, 6.4, and 25.6 mM. In addition, for procaine concentrations of 6.4 and 25.6 versus 0 mM both [Ca²⁺]i and tension were significantly lower at 4°C. As far as the caffeine mentioned above, in Figure 3e,f, we examined the impact of procaine concentration on the temperature thresholds at which high [Ca²⁺]i sites and increased tension were detected. Although compressed necessarily into a narrower range of temperatures than the caffeine responses (i.e., range 8–0°C) note again the close correspondence of the [Ca²⁺]i and tension profiles as increasing procaine concentration from left‐to‐right progressively lowered the temperature thresholds for elevated [Ca²⁺]i and tension. Specifically, compared with the control (0 mM procaine concentration) thresholds of 7.9 ± 1.6°C for [Ca²⁺]i and 11.4 ± 0.7°C for tension, corresponding values for procaine concentration of 1.6 mM were 5.2 ± 1.9°C and ~8.5 ± 0.8, for 6.4 mM, 3.8 ± 2.1 and 4.9 ± 1.2°C, and for 25.6 mM, 1.1 ± 0.5 and 2.2 ± 1.2°C. All of these values, except for the procaine concentration of 1.6 mM tension threshold, were significantly (*p* < 0.05) lower than control.

## 4 DISCUSSION

The present investigation demonstrated the existence of a muscle temperature threshold (approximately 5°C) for in vivo elevation of [Ca²⁺]i and active tension development in response to cooling. This temperature threshold exhibited highly plastic behavior being reduced, in the extreme, to ~2–3°C by the CICR inhibitor, procaine, and increased to ~15–20°C by the CICR promotor caffeine. Since Ca²⁺ signaling is important for various muscle adaptations and homeostasis, cooling‐induced [Ca²⁺]i dynamics in this study may be a novel approach to skeletal muscle adaptation.

We detected a significant heterogeneity in in vivo [Ca²⁺]i response both within and among fibers by means of [Ca²⁺]i imaging (Figure 1a and Video 1) during progressive cooling that induced a muscle contraction. This finding is supported by the previous observation that the caffeine and separately, halothane, concentrations required to produce CICR vary substantially from fiber to fiber (Konishi et al., 1985; Sudo et al., 1990). It is known that there is a temperature distribution in living cells (Okabe et al., 2012), and this may have an effect on skeletal muscle as well. It was notable that the elevated [Ca²⁺]i, followed in real time, appeared to propagate along individual fibers as the cooling progressed. This propagation is similar to the Ca²⁺ wave reported in hypertonic Ringers solution‐induced CICR in frog skeletal muscle fibers (Chawla et al., 2001). The rapid rise in [Ca²⁺]i and increased tension detected herein (Figure 1b) has also been reported in in vitro single muscle fiber experiments (Konishi et al., 1985). As for in vitro single fibers of the frog and other species including mammals, the present investigation reveals that the characteristic increase in [Ca²⁺]i occurs in vivo only on cooling and disappears when normothermia is restored, indicating that it can be regulated by temperature changes and is readily reversible upon removal of the hypothermic stimulus (Figure 1).

Differences in CICR sensing, and therefore [Ca²⁺]i response, among muscle fibers due to cooling may be related to the difference in Ca²⁺ handling as this corresponds to muscle fiber type. For instance, the spinotrapezius muscle is comprised of a mosaic of slow (Type I)‐ and fast(Type II)‐twitch fibers with ~52% IIb and IIx fibers (Delp & Duan, 1996). In a comparison of excised rat soleus and EDL muscles, cooling‐induced tension exertion is detected at a higher temperature range in the soleus muscle (Hill, 1972), suggesting that Type I fibers are more sensitive to cooling. The most quantitatively important SR Ca²⁺‐binding protein in skeletal muscle is calsequestrin (CSQ) (MacLennan & Wong, 1971; Murphy et al., 2009). The rat Type II EDL contains ~4‐fold higher CSQ1 than the Type I soleus muscle. This CSQ1‐associated Ca²⁺ constitutes a substantial pool of releasable Ca²⁺ (i.e., ~4× greater in Type II than Type I fibers), while facilitating maintenance of a very low free SR [Ca²⁺]i which limits SR Ca²⁺ leakage via SERCA (Murphy et al., 2009). As CSQ1 is low in Type I fibers, there is a higher ratio of endogenous to maximal Ca²⁺ content which does not lead to metabolically compromising SR Ca²⁺ leaks, in part, because SERCA1 is essentially absent in Type I muscle fibers; at least in the soleus (Murphy et al., 2009). It is likely that the presence of a relatively larger pool of free SR Ca²⁺ in Type I fibers combined with the extremely low SERCA1 levels may predispose these fibers to greater cooling‐induced Ca²⁺ accumulation and contracture. Unfortunately, in the present investigation, it was not technically feasible to identify individual muscle fiber types in vivo. Thus, within the spinotrapezius muscle in vivo the extent to which the cooling temperature threshold is muscle fiber type dependent remains to be verified experimentally.

In the present investigation, cooling‐induced [Ca²⁺]i elevation was observed at about 5°C with associated muscle contraction under the in vivo environment and in the absence of caffeine or other potentiators. However, an important caveat here is that there may be an impact of general anesthesia. For instance, isoflurane enhances CICR in skeletal muscle (Matsui et al., 1991). Although the possibility that changes in homeostasis due to anesthetics could not be eliminated during these in vivo experiments, the changes in the CICR temperature threshold (i.e., that necessary to produce elevated [Ca²⁺]i and active tension) during cooling induced by caffeine (increased temperature threshold) and procaine (decreased temperature threshold) were statistically significant and substantial in magnitude (Figures 2 and 3). Thus, the results of the present investigation are in substantial agreement with those of Konishi et al. (1985) who found that, in frog skinned single fibers, cooling‐induced muscle contraction in vitro was potentiated with elevated [Ca²⁺]i in a caffeine concentration‐dependent manner. Recent discovery of the caffeine‐binding region of RyR1 by des Georges et al. (2016) has revealed the close spatial proximity of caffeine and Ca²⁺ binding sites on RyR1. Thus, by caffeine binding to the RyR1 channel, its opening probability is enhanced increasing SR Ca²⁺ release and illuminating the mechanistic basis for caffeine increasing RyR1’s Ca²⁺ sensitivity (Ogawa, 1994) and left‐shifting the force‐[Ca²⁺]i curve toward lower [Ca²⁺]i (Wahr & Metzger, 1999). This phenomenon explains how caffeine elevates the temperature threshold at which cooling increases muscle tension. In direct contrast to caffeine, in vitro procaine reduced the [Ca²⁺]i increase and tension developed during cooling‐induced contractions in a concentration‐dependent manner (Konishi et al., 1985). This procaine effect was apparent in the in vivo environment of the present investigation with procaine inhibiting the cooling‐induced [Ca²⁺]i elevation and reducing tension; effectively lowering the temperature required to promote high [Ca²⁺]i and associated tension increases (Figure 3). The decrease in tension with decreasing temperature, which is more pronounced during procaine loading, seems to be different from the [Ca²⁺] dynamics. Although the direct relationship is not clear, it should be noted that the influence of caffeine and procaine on the threshold temperature may differ for [Ca²⁺]i and tension, since temperature reduction decreases Ca²⁺ sensitivity of myofibers (Talon et al., 2000) and also changes the cross‐bridge states (Caremani et al., 2019). However, an important finding of the present study is that interventions that impact CICR control in skeletal muscle can modulate the temperature responsivity to cooling‐induced elevations of [Ca²⁺]i and tension in vivo.

Calcium signaling is involved in a variety of muscle adaptations, including myogenesis, growth and regeneration (Gehlert et al., 2015; Tu et al., 2016). Whether there is a realistic potential to utilize the cooling‐induced increase in [Ca²⁺]i shown in the present investigation therapeutically is uncertain at this time. Transcutaneous cooling of human muscles to temperatures that cause an increase in [Ca²⁺]i is possible but would be extremely challenging. However, our data substantiate that, under in vivo conditions, interventions that promote CICR, such as caffeine, may effectively raise the temperature threshold sufficiently to facilitate practical application of muscle cooling via Ca²⁺ signaling‐induced effects. Caffeine constitutes a commonly consumed stimulant and, via safe oral dosing, has a demonstrated, though not unequivocal, capacity to enhance human endurance performance and possibly muscle strength and/or power production (Davis & Green, 2009; Ganio et al., 2009; Spriet, 2014; Warren et al., 2010). These effects are manifested via central nervous system stimulation (Davis & Green, 2009; Kalmar & Cafarelli, 2006; Reggiani, 2020; Spriet, 2014). One paramount concern, however, when utilizing caffeine to promote elevated [Ca²⁺]i as demonstrated herein, for humans, is whether sufficient caffeine could be administered safely to exert the desired effects. For instance, typical ergogenic caffeine dosing is 6–9 mg/kg with plasma concentrations peaking at ~20–70 µM between 45 and 60 min following consumption (Davis & Green, 2009; Ganio et al., 2009; Goldstein et al., 2010; Spriet, 2014; Warren et al., 2010). It is not known whether such a caffeine concentration lowers the cooling‐induced [Ca²⁺]i elevation threshold in humans but there is the possibility that some other form of CICR potentiator may be applied either orally or topically (transcutaneously) to the target muscle(s). What is certain at present is that the oral caffeine dose required to elevate plasma caffeine concentration into the low mM range demonstrated effective for lowering the threshold in the present investigation would likely be toxic to humans (Neyroud et al., 2019).

## 5 CONCLUSION

We report a novel finding that the existence of a muscle temperature threshold (approximately 5°C) for in vivo elevation of [Ca²⁺]i and active tension development in response to cooling. This temperature threshold exhibited highly plastic behavior being reduced, in the extreme, to ~2–3°C by the inhibitor, procaine, and increased to ~15–20°C by the promotor caffeine. This in vivo model may provide data fundamental to developing new skeletal muscle adaptation approaches via cooling‐induced Ca²⁺ signaling.

## CONFLICTS OF INTEREST

The authors declare no conflicts of interest and that the results of this study are presented clearly, honestly, and without fabrication, falsification, or inappropriate data manipulation.

## AUTHOR CONTRIBUTIONS

R.T., D.P. and Y.K. conceptualized and designed the research; R.T. and A.T. performed the experiments; R.T. analyzed the data; R.T., D.P. and Y.K. interpreted the results of experiments; R.T. prepared the figures; R.T., D.P. and Y.K. drafted, edited, and revised the manuscript; R.T., A.T., D.P. and Y.K. approved the final version of the manuscript.

## Supporting information



Video S1
